# Generalized logistic functions in modelling emergence of *Brassica napus* L.

**DOI:** 10.1371/journal.pone.0201980

**Published:** 2018-08-09

**Authors:** Agnieszka Szparaga, Sławomir Kocira

**Affiliations:** 1 Department of Agrobiotechnology, Koszalin University of Technology, Koszalin, Poland; 2 Department of Machinery Exploitation and Management of Production Processes, Section of Quality Management in Agricultural Engineering, University of Life Sciences in Lublin, Lublin, Poland; College of Agricultural Sciences, UNITED STATES

## Abstract

The objective of this study was to determine whether generalized logistic functions (Richards model with time shift) may be used to predict emergence of winter rapeseed (*Brassica napus* L.) after its seed treatment with plant extracts from *Taraxacum officinale* roots under controlled environment conditions. Emergence analyses were conducted for winter rape whose seeds were treated with a plant extract and for the non-treated seeds sown to the soil at the site of earlier point application of the extract. Curves were plotted for experimental data by minimizing the square sum of differences between the experimental data and the mathematical model. To evaluate model fit, the mean squared error was divided into four factors. Computing modelling efficiency coefficients were also introduced to enable complete analysis. Results of simulation research demonstrate that the determined parameters of curves (e.g. values of growth parameters, time shift or the upper limit of population) describing the number of seedlings in the function of time stayed compliant to the interpretation with regard to the biology of the analyzed processes. The proposed mathematical description based on generalized logistic functions showed extraordinary fit (r = 0.999) to the experimental data, which makes it highly useful in predictive control of rapeseed emergence. In addition, the study enabled concluding that plant extracts application to the soil allowed achieving a higher maximal emergence rate compared to the control sample. The application of the plant extracts increased the final population of rapeseed and significantly accelerated the occurrence of the maximal emergence rate.

## Introduction

The emergence of seedlings is probably the most important phenological event that affects the probability of success of plant growth. This success may be boosted by pre-sowing applications of plant extracts of various types that modify the environment around the germinating seeds, as they are rich in bioactive compounds in the form of secondary metabolites that may be subsequently used for plant protection [1–8]. The time at which seedlings appear often affects the competition of a plant with its neighboring plants, its exposure to herbivores or disease infections, and its appropriate maturation before the end of the growing period. Surprising is the fact that plant emergence has not been scrutinized enough so far to enable its reliable growth prediction even for the most popular and important annual species. Studies have relied on the use of non-mathematical methods to predict seedling emergence, including the moment at which 50% of the seedlings have emerged, but these have proven to be unreliable in practice [9]. Alternatives to this case are mathematical methods which allow the prediction of the time at which seedlings appear, which is considered as an element of the integrated crop production management system. The possibility of improving and predicting both germination and emergence is crucial in agricultural practice, as it entails the implementation and optimization of cutting-edge technologies, thus making use of elements of precise agriculture in plant production [10–17]. Two approaches may be observed today in the modelling of seed germination and seedling emergence. The first involves the use of empirical models that are proved accurate in predicting defined results, whereas the second engages models of mechanisms that drive the biological processes [17–18]. Although empirical models have been employed successfully in many studies, little is understood about the biological association or significance of the parameters estimated from the models [19].

Mathematical description of biological growth (i.e. population models) is very important in many research disciplines, including biology, agriculture, and forestry. Among these population models, especially noteworthy are clear analytical solutions of a generalized logistic equation, also known as generalized logistic functions [17, 20–22]. Thus far, these functions have not been used to describe the emergence of plants whose growth environments have been modified, and this makes them potentially interesting in this respect. Considering the above, the objective of this study was to determine whether generalized logistic functions may be used to predict the emergence of winter rapeseed (*Brassica napus* L.) after its seed treatment with plant extracts from *Taraxacum officinale* roots under controlled environment conditions.

## Materials and methods

In this study, we used results elicited from the evaluation of the emergence of winter rapeseed (*Brassica napus* L.) of ‘Sherlock’ cultivar treated with plant extracts prepared from the roots of the common dandelion (*Taraxacum officinale*). The seedling emergence was analyzed in a pot experiment that was established in four replications for each combination. Each combination included 40 seeds (the manuscript presents averaged results from n = 160 seeds). Forty seeds were sown at a depth of 1.5 cm in each pot that was filled with podzolic soil on weak clay sand and gravel ground corresponding to soil class of type IV used for a good rye complex. The experiment was conducted at fixed soil moisture of 80% and ambient temperature of 18°C. The extracts from *Taraxacum officinale* roots was prepared in the form of infusion according to the methodology outlined by Sas-Piotrowska and Piotrowski [23]. Two application methods of water extracts from dandelion roots were used in this study: (1) sowable material was treated by soaking the seeds in water extracts for 24 h, and by air drying them on filter paper and sowing them to the soil, and (2) non-dressed seeds were sown to the soil at the site of point application using 1 mL of the extract. Non-dressed seeds sown to the soil that was not treated with the plant extracts served as the controls. Emerging seedlings were counted each day. The emerging seedlings were counted and marked systematically every 24 h from day 1 to 15 after sowing to determine their germination capabilities and course of plant emergence. The mathematical description of winter rapeseed emergence was conducted with the use of growth functions—generalized logistic functions. The following equation was assumed as a manifestation of the generalized logistic functions (Richard’s functions with time shift),

$${N(t)=K∙[1+Q∙\text{exp}\left(-B∙(t-C)\right)]}^{-v}$$ (1)

where: *t* denotes for time, and *K*, *Q*, *B*, *C*, and ν, are function parameters [17, 21, 24, 25].

Parameter K expresses an asymptotic value of the number of germinations for the time aiming at infinity, Q is a parameter related to the origin of the growth curve N(t), parameter B [1/day] is the growth rate, C [day] is a parameter which is responsible for the temporal shift of the curve, and ν is a parameter responsible for the relative location of the point of inflection of the curve. The choice of units for parameters B and C resulted from the fact of daily measurements of the population size. For the defined functions, a computer model was developed (The following sequence of actions was adopted: Determination of the range of variability of parameters of the searched curve; Generation of sets of parameters—Monte Carlo method; Generation of matrices of dependent variables N(t) for the set parameters; Determination of the explicit form of the searched curve) in MATLAB (version 2016). This model enabled the determination of the parameter values of the analyzed functions for which the square sum of the differences between the predicted model values and experimental data was assumed to be minimized.

To analyze the quality of fit of the obtained curves and for comparative purposes of the two tested models, two rates were introduced, namely, the mean square error Δ^2^ and a normalized value of the sum-of-squares of deviations Δ^2^_norm_ in the form of

$$\Delta^{2}=\sum_{i=1}^{n}{\left(N_{i}^{theor}-N_{i}^{exp}\right)}^{2},n=16,N_{i}^{exp}=N^{exp}(t_{i})$$ (2)

$$\Delta_{norm}^{2}=\frac{1}{N_{max}}\sum_{i=1}^{n}{\left(N_{i}^{theor}-N_{i}^{exp}\right)}^{2},N_{max}=\underset{i}{\text{max}}\;N_{i}^{exp}$$ (3)

where n = 16 and stands the number of measurement points (1 readout/day for 15 days), *N*_*i*_^*exp*^ and *N*_*i*_^*theor*^ are respectively the measured and calculated values of the emergence percentage within the *i*^*th*^ -time instant (*t*_*i*_ = *{1*, *2*,…, *15}* days), and *N*_*max*_ is the maximum value of the germination percentage in the investigated period.

The value of a correlation coefficient was also introduced in accordance to

$$r=\frac{n\sum_{i=1}^{n}(N_{i}^{theor}∙N_{i}^{exp})-\sum_{i=1}^{n}N_{i}^{theor}∙\sum_{i=1}^{n}N_{i}^{exp}}{\sqrt{\left[n\sum_{i=1}^{n}{\left(N_{i}^{theor}\right)}^{2}-{\left(\sum_{i=1}^{n}N_{i}^{theor}\right)}^{2}]∙[n\sum_{i=1}^{n}{\left(N_{i}^{exp}\right)}^{2}-{\left(\sum_{i=1}^{n}N_{i}^{exp}\right)}^{2}\right]}}$$ (4)

The error of a model ought to be evaluated in terms of two traits, namely, deviation and precision. The first refers to the deviation of the mean of model errors from zero, whereas the other refers to the extent of model errors. Obviously, both traits must be analyzed to evaluate the model’s efficiency. The error and precision may be evaluated using statistical estimators [26]. Model precision was evaluated based on the determined mean squared prediction error,

$$mse=\frac{1}{n}\sum_{i=1}^{n}{\left(N_{i}^{theor}-N_{i}^{exp}\right)}^{2}$$ (5)

Based on a work by Gauch et al. [27], the mse was expressed as the summation of two terms,

mse=sb+nu+lc (6)

where

sb denotes the squared bias

$$sb={\left(\frac{1}{n}\sum_{i=1}^{n}N_{i}^{exp}-\frac{1}{n}\sum_{i=1}^{n}N_{i}^{theor}\right)}^{2}$$ (7)

nu denotes non—unity slope

$$nu={\left(1-b\right)}^{2}\frac{\sum_{i=1}^{n}{\left(N_{i}^{theor}-\frac{1}{n}\sum_{i=1}^{n}N_{i}^{theor}\;\right)}^{2}}{n}$$ (8)

and lc denotes the lack of correlation

$$lc=(1-r^{2})\frac{\sum_{i=1}^{n}{\left(N_{i}^{exp}-\frac{1}{n}\sum_{i=1}^{n}N_{i}^{exp}\;\right)}^{2}}{n}$$ (9)

In addition, a comparative analysis was conducted between the predicted and observed data using statistics analogous to R^2^, sometimes referred to as the modelling efficiency (ef):

$$ef=1-\frac{\sum_{i=1}^{n}{\left(N_{i}^{exp}-N_{i}^{theor}\right)}^{2}}{\sum_{i=1}^{n}{\left(N_{i}^{exp}-\frac{1}{n}\sum_{i=1}^{n}N_{i}^{theor}\right)}^{2}}$$ (10)

This statistic indicators is a simple indicator of efficiency on a relative scale, where 1 denotes a “perfect” fit, 0 indicates that the model is not better than the arithmetic mean, whereas negative values point to a poor fit of the model to the observed results.

## Results and discussions

**Fig 1:**
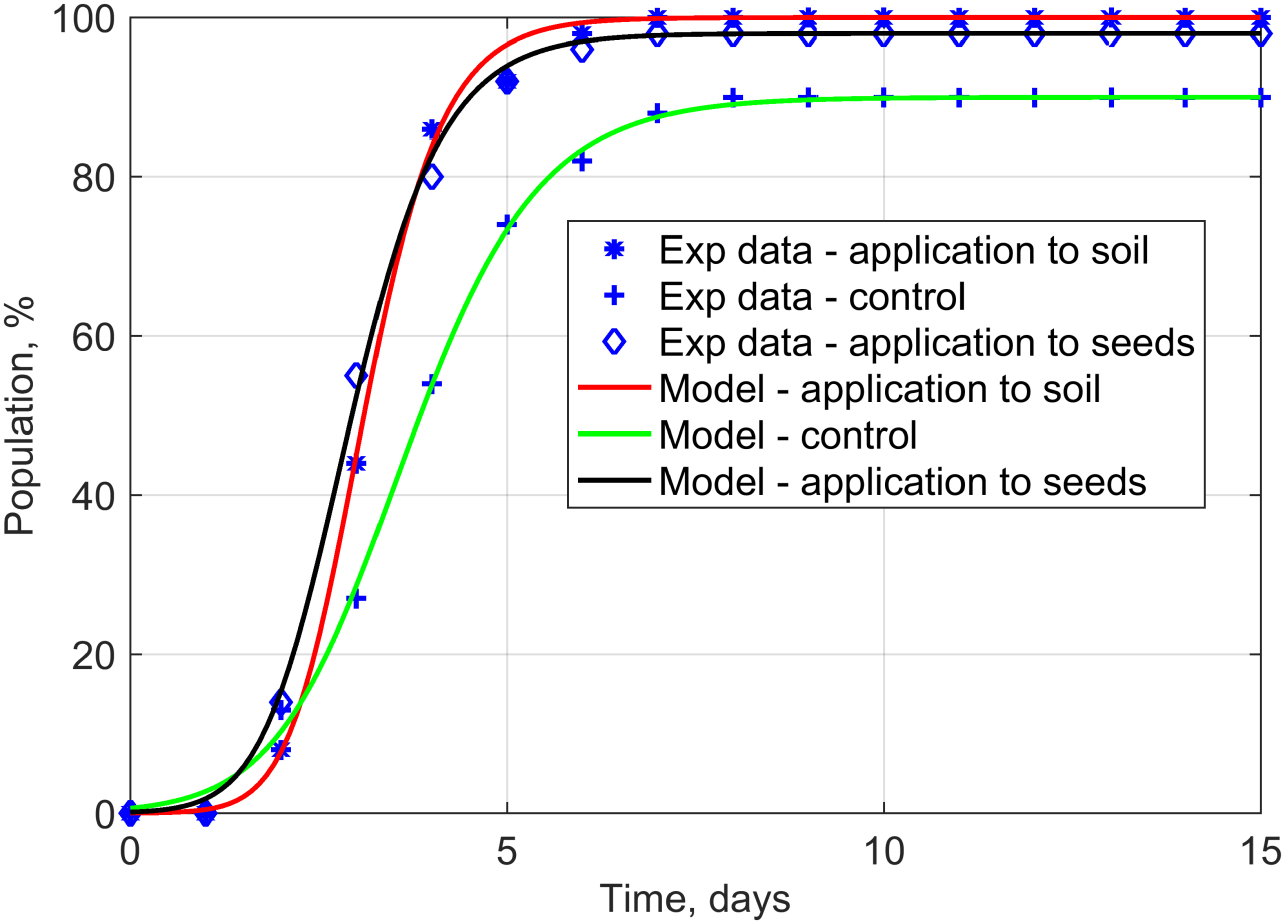
Time relations of emergence of rapeseed seedlings for non-treated seeds (control) and for two methods of application of plant extracts (to soil and on seeds)—Generalized logistic curves.

With the use of the developed computer model, the generalized logistic curves were determined, which defined the time evolution of the germination of rapeseed seedlings for non-dressed seeds (controls), seeds treated with plant extracts, and seeds after in-soil application of extracts (Fig 1).

The conducted evaluation of model precision and computing modelling efficiency (Table 1) demonstrated that the proposed mathematical description based on generalized logistic functions yielded an extremely good fit (r = 0.999, ef = 0.998) to the collected experimental data, which makes it highly useful in the predictive control of rapeseed emergence.

**Table 1:** Evaluation of model precision and computing modelling efficiency for rapeseed seedlings for non-treated seeds (control) and two methods of application of plant extracts (to soil and on seeds).

Analyzed combination	Generalized logistic model—Richards model with time shift							
	r	mse	sb	nu	lc	ef	Δ^2^	$\Delta_{norm}^{2}$
**Control**	0.9995	1.3310	0.0027	0.0679	1.2605	0.9989	21.2785	0.2364
**Application to soil**	0.9994	1.8242	0.0835	0.0091	1.7316	0.9987	29.1880	0.2919
**Application on seeds**	0.9995	1.5158	0.1306	0.0287	1.3565	0.9988	24.2478	0.2474

**Fig 2:**
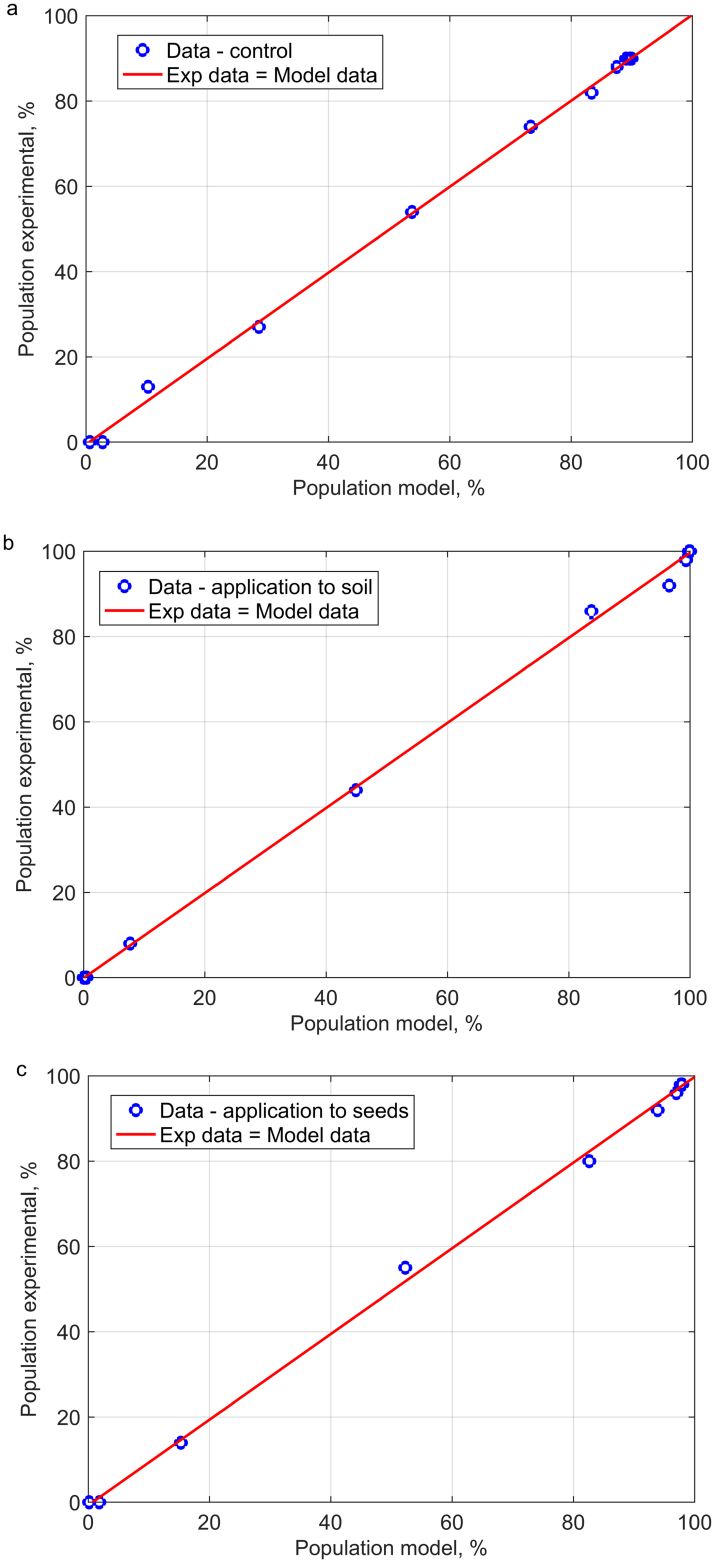
Correlation between the experimentally determined emergence percentage and respective predicted values emergence of winter rape for control (a) and two methods of application of plant extracts to soil (b) and seeds (c).

The determined components of the mean squared prediction error (Eqs 5 and 6) can be interpreted in a simple geometric manner in the case of the analysis of a correlation between the experimentally determined emergence percentage and the respective predicted values (*N*_*i*_^*exp*^ *vs*. *N*_*i*_^*theor*^) (Fig 2). The straight line crossing the onset of the coordinate system with a slope of 45° corresponds to the case where mse = 0 (the perfect line). In the case where mse>0 the following may result: a) line translation when sb> 0,a change of the slope angle when nu>0 and scattering of individual points when lc>0.

The generalized logistic model designed for the controls was characterized by the lowest values of the mean squared prediction error. However, the sources of deviations were different for each analyzed rapeseed combination. In the case of the on-seed application of the plant extracts the value of the squared bias was the highest, which was observed as a vertical translation of the perfect line fit (*N*_*i*_^*exp*^ *vs*. *N*_*i*_^*theor*^). The value of the non-unity slope indicator responsible for the rotation of the straight line was the lowest in the case of rapeseed emergence in combination with the in-soil applications of plant extracts. The scattering of individual points expressed by the value of the lack of the correlation coefficient was the smallest in the case of the correlation between the experimental and predicted results for the emergence of winter rape seedlings in the case of non-dressed seeds (control).

**Fig 3:**
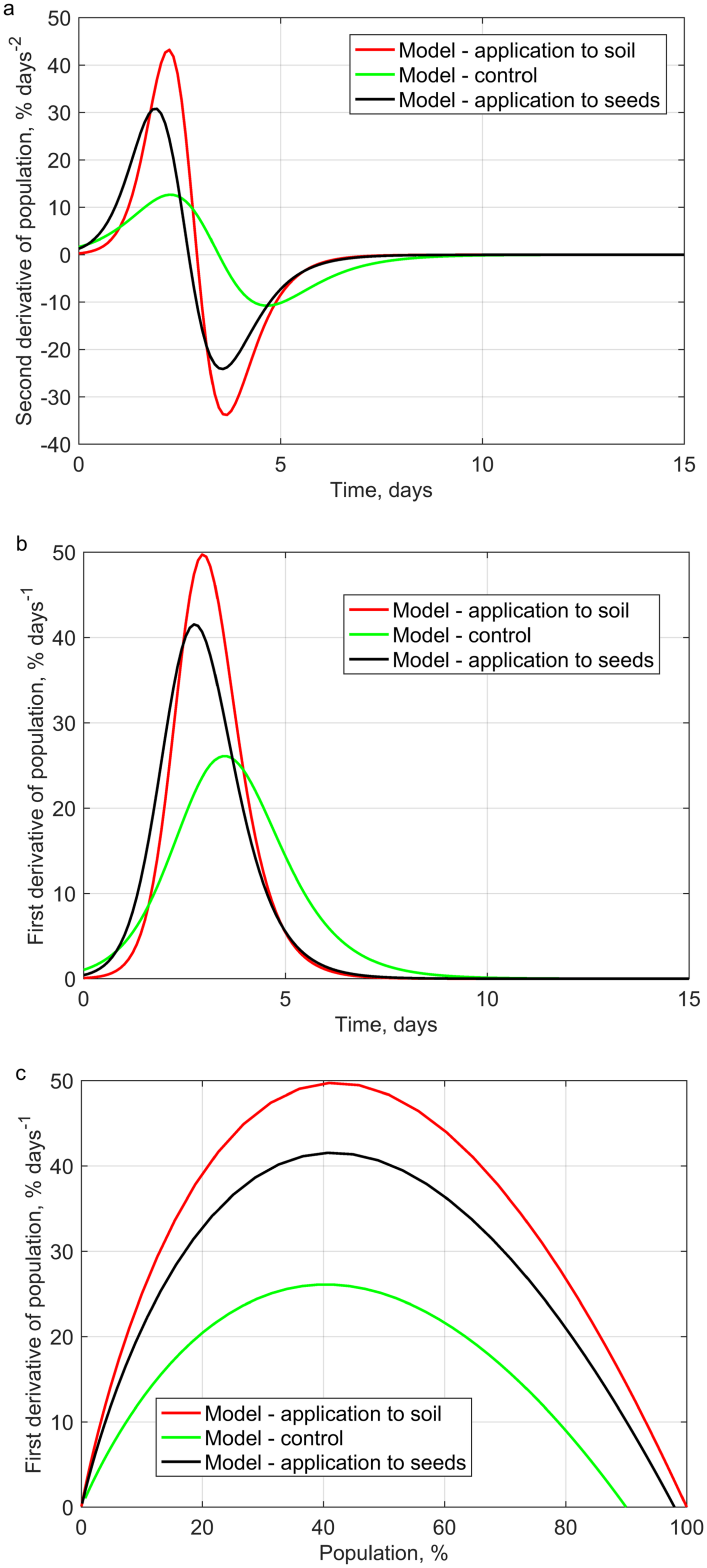
Time dependence of a) first and b) second derivative and c) phase portrait of the generalized logistic curves (Fig 1).

Furthermore, the time courses of the first and second derivatives as well as the phase portrait (Fig 3a–3c) were determined for the analyzed generalized logistic functions (Fig 1). The analysis of the phase portrait (Fig 3c) shows the asymmetry of phase trajectories that are typical of the Richard’s model. This means that unlike the Verhulst model, the time needed to reach the highest value of growth rate differs from the time after which the population reaches half the maximum value. The highest seedling growth rate was recorded between days 3 and 4. However, in the case of applications of extracts from dandelion roots, the highest growth rate was observed before the 3^rd^ day of the experiment. The exact times at which maximum seed derivatives occurred were at 2.95 d and 2.75 d for in-soil and on-seed applications of the plant extracts, respectively (Fig 3). Analyses of the emergence of the non-dressed seeds demonstrated that the highest seedling growth rate occurred later compared to the cases of application combinations with plant extracts (3.5 d). The application of the plant extracts achieved a greater final population of rapeseed and significantly accelerated the occurrence of the maximum growth rate of seedlings (Fig 3).

The logistic generalized functions are suitable for the predicting emergence in the studies with seeds treated with plant extracts. In our earlier study generalized logistic functions and Koya-Goshu functions were used to describe the time evolution of germination of beetroot seedling for combinations of non-dressed (control) seeds dressed with extracts, and for seeds sown to soil treated with extracts. Outcomes demonstrated that for the control, the Koya-Goshu model yielded an almost two-fold fit improvement to experimental results in comparison to the generalized logistic model. In the case of on-seed application of plant extracts, the generalized logistic model yielded a five-fold improvement regarding the fit to the experimental results [17].

Among many models described in the literature, special attention has been paid to the analytical solutions of the generalized logistic equation, commonly reffered to as generalized logistic functions.

The so-called competition function is part of the Verhulst model (which describes the competition for natural resources), which ensures that, at specified period of time, a population reaches the established K value which represents the upper limit of the growth size. Introduction of this function to the growth equation allows the establishment of a realistic exponential model that is characterized by the unlimited growth in a longer period of time [21].

By using the analogy of germination processes to the growth processes of any system objects described with a logistic function, Gładyszewska [28] postulated a model which may be used to the describe germination curves plotted for seeds of various plants. In addition Pietruszewski [29] discussed the feasibility of modelling wheat seed germination based on the logistic curve. The analysis of plant germination under diversified environmental conditions is a crucial area in agricultural and ecological surveys [30].

Berry et al. analyzed the possibility of describing a germination curve with a generalized Richard function [31]. Obtained results demonstrated that the values of parameters describing the shapes of the germination curves plotted for the seeds of most of the analyzed crops were identical as these were computed using the Gompertz or the logistic functions [12].

Based on the results in our study, it was concluded that generalized logistics functions may be useful in predicting the emergence of winter rape (*Brassica napus* L.) after the application of plant extracts from roots of *Taraxacum officinale*. The model presented in our study was used to analyze the growth of rapeseed under controlled laboratory conditions when the air temperature and soil moisture content were kept at stable and optimal levels for plant growth. Under natural conditions, however, the main factors which regulate seed germination and plant emergence include temperature, water potential of the environment and air quality [32–34]. Germinating seeds are characterized by increased sensitivity to thermal and hydrological conditions and by a strong and rapid response to any external physical factors. Hence, the ability to predict the time of seedlings emergence seems to be an element of the integrated system for crop production management.

Hydrothermal models underlay the recently undertaken efforts aimed at predicting seed germination. Their key feature is the assumption that each of the seeds accumulates hydrothermal time depending on the temperature and water potential compared to the base temperature and water potential, thus enabling seed development. Their main advantage is that their equations are suitable for the entire population of seeds and allow for the simultaneous prediction of the germination rate and percentage of germinating seeds [35–38]. It is often unclear how data from experiments conducted under controlled conditions will correspond to data gathered from field experiments performed under varying environmental conditions. Therefore, based on results obtained in our experiment, we have presented hypothetical considerations on the potential evolution of the determined curves in accordance with field conditions.

**Fig 4:**
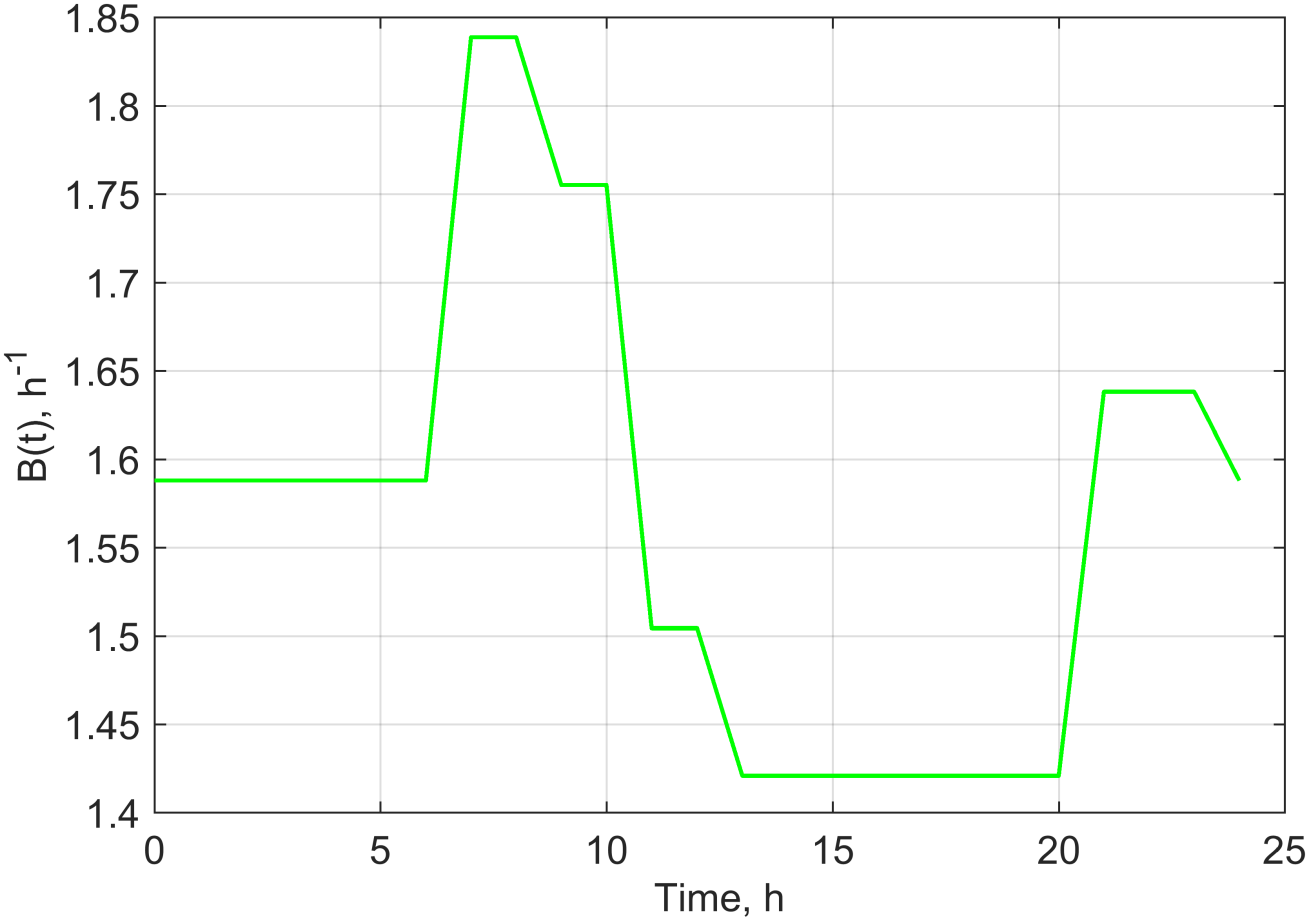
Time dependence of B(t) used in scenario 1.

**Fig 5:**
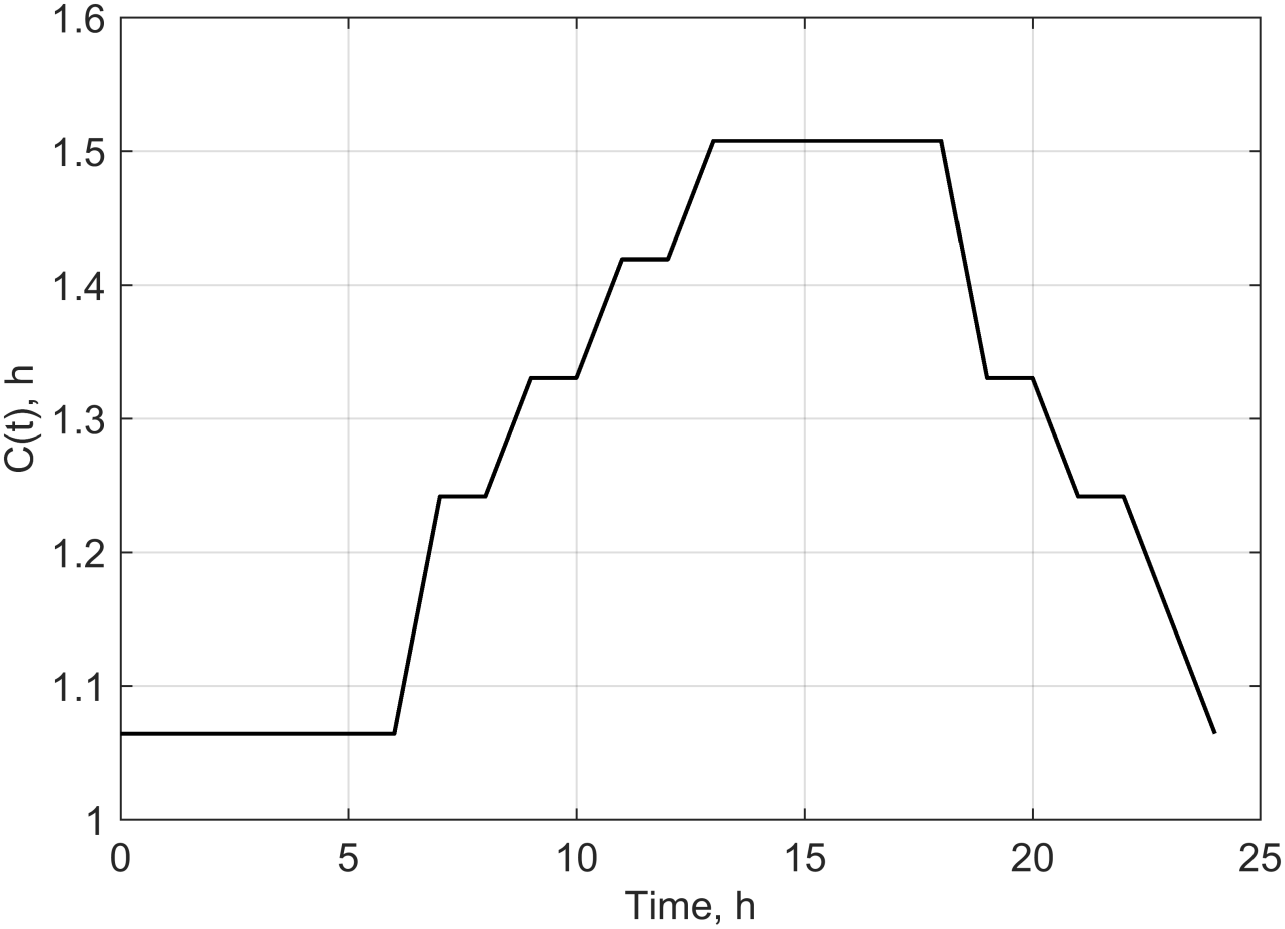
Time dependence of C(t) used in scenario 2.

**Fig 6:**
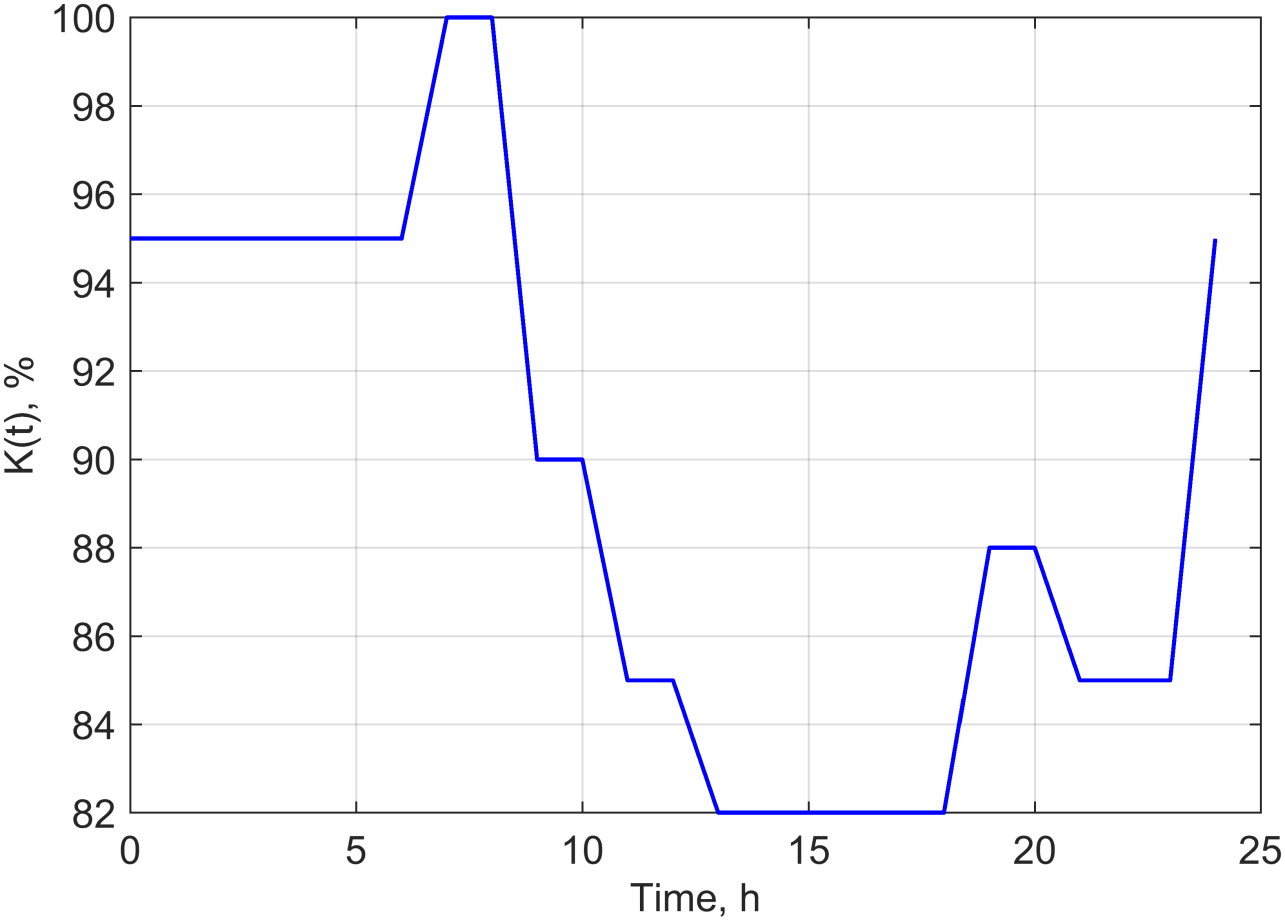
Time dependence of K(t) used in scenario 3.

However the potential scenarios proposed will be verified in our future experimental research. We assumed in the study conducted under controlled conditions that values of the growth coefficient, environment capacity and time shift would be stable. However, an attempt for transferring the model developed based on a laboratory experiment into real field experimental conditions has prompted us to consider that these parameters are also dependent on temperature, air humidity and field water capacity. These constants were not forced to be explicitly dependent on temperature, humidity and water capacity, but through periodical change in their values. The periodical changes (within 1 day) of the parameters: B(t), C(t) and K(t), proposed based on average weather data for the area of Poland [39] are presented in Figs 4–6.

In successive simulations we have assumed that a daily change in a given set of parameters (Figs 4–6) would repeat every other day throughout the analyzed time period. By using the proposed dependencies of B(t), C(t) and K(t) (Figs 4–6), we have modified the periodical evolution of the population under laboratory conditions for the three analyzed scenarios according to the following functions (Eqs 11–13).

$$N(t)=0.95K_{lab}∙{\left[1+Q_{lab}∙exp(-B(t)∙(t-C_{lab}))\right]}^{{-v}_{lab}}$$ (11)

$$N(t)=0.9K_{lab}∙{\left[1+Q_{lab}∙exp(-B_{lab}∙(t-C(t)))\right]}^{{-v}_{lab}}$$ (12)

$$N(t)=K(t)∙{\left[1+Q_{lab}∙exp(-B_{lab}∙(t-1.5C(t)))\right]}^{{-v}_{lab}}$$ (13)

where *K*_*lab*_, Q_lab_, C_lab_, B_lab_, and ν_lab_, denote the values of constants obtained from the model fitting to the experimental data under controlled conditions for the treated soil, whereas N_1_(t), N_2_(t) and N_3_(t), indicate the population sizes in the hypothetical scenarios 1 (Eq 11), 2 (Eq 12), and 3 (Eq 13).

**Fig 7:**
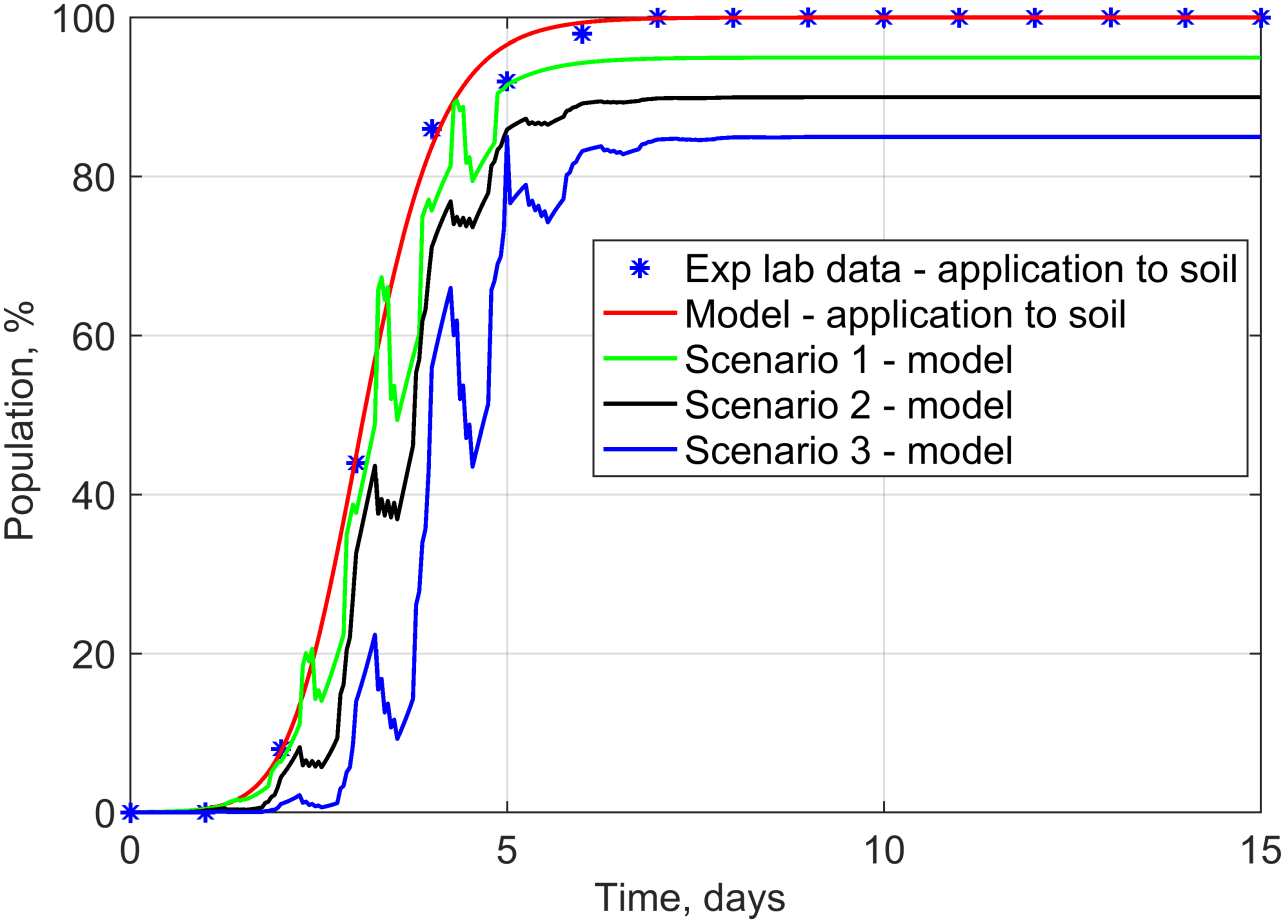
Time dependence of emergence of rapeseed seedlings for the application of plant extracts to soil (laboratory experiment) and for virtual scenarios 1–3.

Population time courses obtained for the analyzed scenarios together with real experimental data achieved after the applications of plant extracts to the soil are depicted in Fig 7.

The analysis of the obtained results enables the conclusion that a change in theparameter B(t) in scenario 1 evokes cyclical, local changes in population monotonicity, which may represent changes in the activity of the plant’s photosynthesis process. In the case of scenario 2, a change in C(t) leads to local inhibitions of population growth, which may represent, among other things, fluctuations in daily courses of temperature and soil humidity. A change in K(t) and C(t) in scenario 3 leads to more abrupt changes in the temporal dependence of the population compared to the other scenarios and is owing to the synergy of changes in the environmental capacity and time shift. This scenario may describe a situation when rapeseed plants are exposed to strongly varying meteorological conditions and locally changing conditions of the biotope.

Simulations of potential changes in the constants of the generalized logistic model that is used to analyze the plant development after the treatment with extracts from dandelion, would enable advances in the development of hydrothermal threshold models. The latter will in turn allow more accurate predictions of seed behavior in real environments [40]. Today, the most promising concept in modelling is the “hydrotime” concept. Its idea was first presented and explained by Gummerson [41], and was subsequently investigated and extended by Bradford [34]. Until the present time, this concept has been mainly exploited to describe seed germination under laboratory conditions and not seedlings emergence under real field conditions. Nevertheless, the hydrotime idea coupled with thermal time was extended in the form of the concept of “hydrothermal time” and has proved to be considerably so attractive that it is envisaged to develop rapidly in the future.

Summarizing the need for improving the existing plant growth models, consideration should be given to the feedback between conditions occurring in a given area and values of parameters describing population growth. The presented hypotheses concerning the possibility of transferring developed models from conditions of a controlled environment to real-field conditions indicate the direction of future studies. Elicited results from these studies may provide a significant input to the mathematical description of crop development.
